# Electrolytic Manganese Dioxide Coatings on High Aspect Ratio Micro-Pillar Arrays for 3D Thin Film Lithium Ion Batteries

**DOI:** 10.3390/nano7060126

**Published:** 2017-05-27

**Authors:** Yafa Zargouni, Stella Deheryan, Alex Radisic, Khaled Alouani, Philippe M. Vereecken

**Affiliations:** 1 KACST-Intel Consortium Centre of Excellence in Nano-Manufacturing and Applications (CENA), Riyadh 11442, Saudi Arabia; 2 Imec, Kapeldreef 75, B-3001 Leuven, Belgium; stella.deheryan@jsrmicro.be (S.D.); alex.radisic@imec.be (A.R.); philippe.vereecken@imec.be (P.M.V.); 3 Laboratoire de Chimie Analytique et D’électrochimie, Faculté des Sciences de Tunis, Université de Tunis-El-Manar, Tunis 2092, Tunisie; khaled.alouani@gmail.com; 4 KU Leuven, Centre for Surface Chemistry and Catalysis, Faculty of Bioscience Engineering, Kasteelpark Arenberg 23 bus 2461, B-3001 Leuven, Belgium

**Keywords:** electrodeposition, EMD, thin film, carbon, 3D current collector, microbatteries

## Abstract

In this work, we present the electrochemical deposition of manganese dioxide (MnO_2_) thin films on carbon-coated TiN/Si micro-pillars. The carbon buffer layer, grown by plasma enhanced chemical vapor deposition (PECVD), is used as a protective coating for the underlying TiN current collector from oxidation, during the film deposition, while improving the electrical conductivity of the stack. A conformal electrolytic MnO_2_ (EMD) coating is successfully achieved on high aspect ratio C/TiN/Si pillar arrays by tailoring the deposition process. Lithiation/Delithiation cycling tests have been performed. Reversible insertion and extraction of Li^+^ through EMD structure are observed. The fabricated stack is thus considered as a good candidate not only for 3D micorbatteries but also for other energy storage applications.

## 1. Introduction

**Scheme 1:**
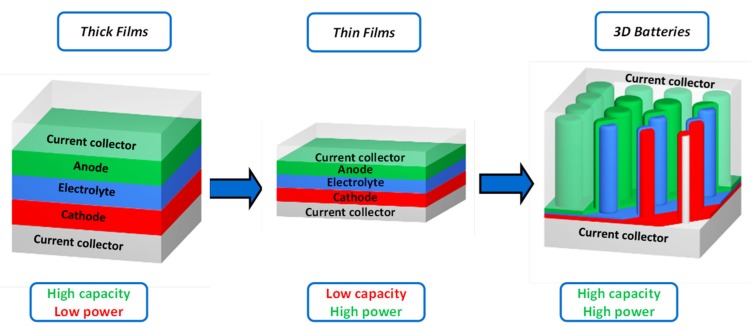
Illustration showing the evolution from planar thin-film batteries with 1 to 2 μm thick electrode films to 3D thin-film batteries with sub-micrometre thin films.

In the last few years, there has been a growing interest in the improvement of power supply sources to satisfy the increasing demands for energy [1]. Among the conventional energy storage systems, lithium-ion batteries (LIBs) are considered state-of-the-art technology for application in smart electronic devices due to their high energy density [2,3]. In this regard, considerable attention has been paid to the development of lithium ion microbatteries in both thick and thin film forms [4]. However, the planar form of the current thin-film batteries provides only limited capacity (<1 mAh/cm^2^) which restricts their use to the low power applications. In spite of the achievements in the optimization of thin film microbatteries designs, their limitations motivate researchers to find new alternatives [4]. Therefore, new approaches to electrode design have been identified to achieve fast battery charging combined with high energy density. In this respect, micro-fabrication and/or nano-structuring of three-dimensional (3D) current collectors with high aspect ratios provide enlarged surface area [5]. This results in the quantitative increase of the deposited active material while keeping the thin film assembly to provide short ion diffusion paths and good electron harvesting pathways (Scheme 1) [6,7,8,9,10]. 

Several methods for 3D electrodes manufacturing have been reported in the literature [7,8,9,11,12]. Silicon (Si) is widely used as processing material for electronic devices integration [13,14]. Hence, Si patterning electrochemically or via deep reactive ion etching (DRIE) techniques has led to high aspect ratio structures, for microbatteries and micro-electro-mechanical-systems (MEMS) applications [15,16,17]. Usually, Si microstructured substrates require a barrier diffusion layer of titanium nitride (TiN) or tantalum nitride (TaN) to prevent Li ions penetration into Si. Such thin film also ensures the electrical contact and is considered as current collectors owing to their sufficient conductivity [18,19,20].

Three-dimensional electrode architectures have been proposed so far in numerous research works [15,21,22]. For example, micro-channels through Si as well as deep trench architectures have been reported [17,23]. Nanowire arrays and nanorods have been also detailed showing an improvement in electrode performance due to the current collector geometries as well as the deposited active materials like Cu_2_Sb, Fe_2_O_3_ and TiO_2_ [7,24,25,26,27]. Such materials can be prepared by a variety of techniques such as electrochemical deposition (ECD) [12,28]**,** atomic layer deposition (ALD) [29,30], chemical vapor deposition (CVD) [31], and sol-gel deposition [9]. For 3D current collectors, the deposition of a conformal coating is challenging. To date, ALD is considered the common technique to make a conformal coating on high aspect ratio structures [32]. However, it needs expensive precursors and the deposition time is relatively long [31,33]. In contrast, ECD is a cost-effective method that allows a conformal deposition of the active material over 3D complicated structures [8].

Since the 1990s, manganese dioxide (MnO_2_) has been considered promising material for batteries [34,35] for its low cost, non-toxicity, natural abundance of Mn and the ease of manufacturing [36,37,38]. The Li/MnO_2_ battery system is known as a primary cell with an operating voltage of 3 V and a MnO_2_ electrode capacity up to 250 mAh/g [39,40,41].They are also widely utilized in capacitors [42], hybrid(asymmetric) capacitors [43], supercapacitors [44] and electrocatalysis applications [45,46]. Among the different polymorphs of this metal oxide, γ-MnO_2_ is the most electrochemically active form [47]. It is mainly produced by ECD and called electrolytic manganese dioxide (EMD) [40]. γ-MnO_2_ is described as an intergrowth between Pyrolusite and ramsdellite-forms of MnO_2_ [27]. The as-prepared EMD contains water in its film structure [48]. In order to enable reversibility and avoid lithium hydroxide formation upon Li-ion intercalation tests, removal of water from EMD thin films is necessary. Therefore, post deposition annealing between 250 and 400 °C is essential [49]. This heat treatment leads to an enhanced lithium diffusivity in the electrode accompanied with improved crystallization and grain growth [48,49,50]. Previous studies reported the deposition of EMD on Si substrates coated with TiN and Pt seed layers as current collectors. It was revealed that TiN gets oxidized during EMD formation and the TiON*_x_* passivating layer prevents much more growth of the deposited film [10,51,52]. In the case of Pt current collector, EMD films delaminated when reaching a thickness more than 200 nm because of the poor adhesion between Pt and EMD [51]. Recently, we demonstrated that a carbon coating, grown by plasma enhanced chemical vapor deposition (PECVD), on TiN protects it from oxidation and enables the deposition of up to 500 nm EMD film. The carbon buffer layer acts as a protective coating, enhances the adhesion between TiN and EMD and improves the electrical conductivity of the stack. Thus far, only results for planar C/TiN/Si substrates were reported [52]. 

**Scheme 2:**
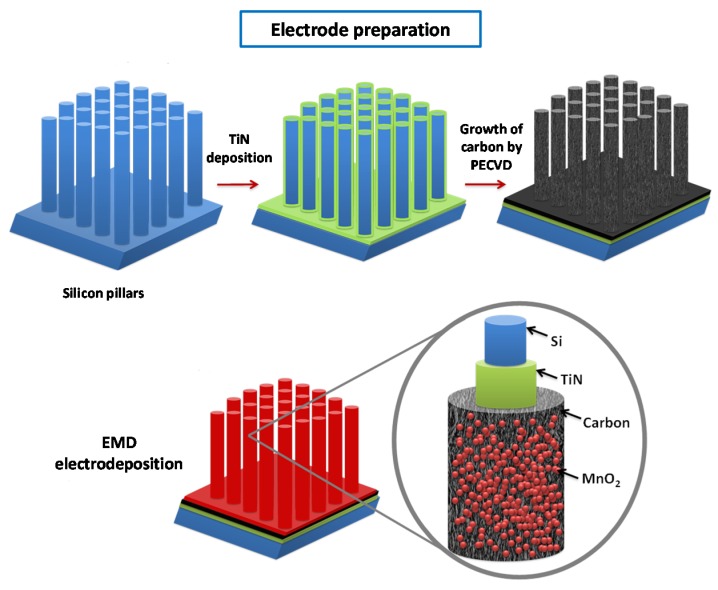
The steps of electrolytic manganese dioxide (EMD) thin film electrode preparation on C/TiN/Si pillars substrate from an aqueous bath of MnSO_4_ in H_2_SO_4_. More details will be provided in the experimental section.

In this paper, we study the deposition of EMD coatings on high aspect ratio (60:2) TiN/Si pillars. Prior to film deposition, the 3D substrate was coated with the PECVD carbon layer, (Scheme 2). The use of carbon coating, as well as the morphology of EMD on improving the reversible Li-ion intercalation kinetics for the assembled electrode, will be examined [52,53]. The influence of the bath temperature, from which the film was deposited, as well as the impact of film thickness, are also investigated. 

## 2. Experimental

### 2.1. Fabrication of Silicon Pillar Arrays

**Figure 1:**
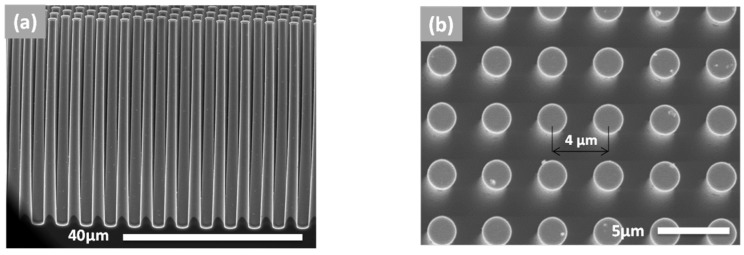

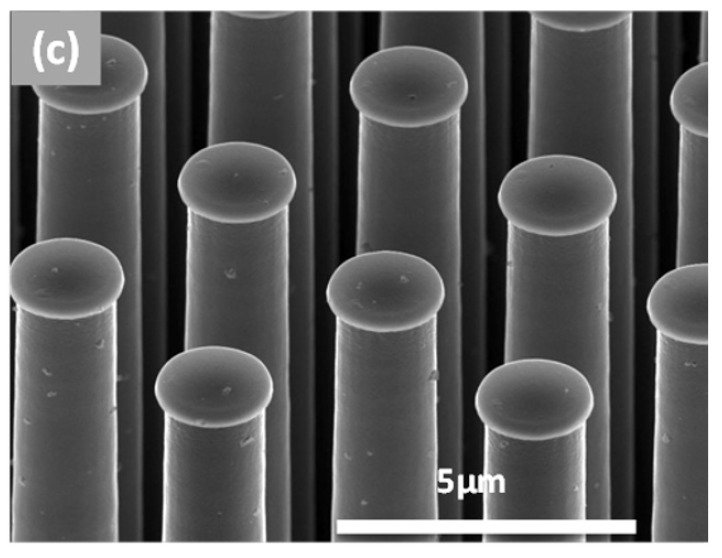
(**a**) Cross-section, (**b**) top and (**c**) tilted view SEM images for TiN coated silicon pillar arrays.

Square arrays of silicon micro-pillars 2 μm in diameter with 2 μm spacing (or 4 μm inter-pillar distance) (Figure 1a,b) were prepared by the Deep Reactive Ion Etching (DRIE) process [54,55]. A 600 nm SiO_2_ hard mask was used for 55–60 μm deep etches. The remaining hard mask after DRIE can be seen on top of the pillars in Figure 1c. 

Afterwards, a dry etch step was done to remove the polymeric residues of the DRIE process from the pillar side walls. Then, the obtained Si pillars were coated with 20 nm TiN film by atomic layer deposition (ALD). These 55–60 μm high pillars give an aspect ratio of 30 with an area enhancement of about 25×.

### 2.2. Carbon Coating on TiN/Si Pillars

**Figure 2:**
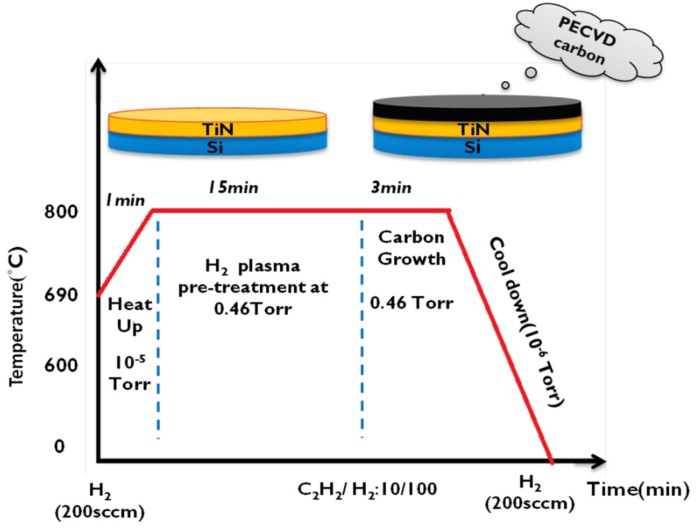
Representation of the process of PECVD carbon deposition and its profile. In this graph, the experimental parameters (Gas flow, temperature setting) are diagrammatically shown.

The TiN/Si pillars were subsequently coated with 30–50 nm carbon films [56,57,58]. The carbon deposition is conducted in NANOCVD, a capacitively coupled PE-CVD reactor with a 13.56 MHz Radio Frequency (RF) generator (Oxford Instruments, Bristol, UK). The PE-CVD process was carried out using a C_2_H_2_:H_2_ (10:100) mixture at low pressure (0.46 Torr) and at a temperature of 800 °C. The carbon coating was grown on 20 nm ALD TiN coated silicon pillars as illustrated in Figure 2. The TiN layer enables the electrical contact for the PE-CVD carbon and serves as a lithium diffusion barrier for the underlying Si [51,57].

### 2.3. Deposition of EMD Thin Films on Carbon Coated TiN/Si Pillars

EMD thin films were anodically deposited from an aqueous bath of (0.3 M) MnSO_4_·H_2_O (98.0–101.0%, Alfa Aeser, Karlsruhe, Germany) and (0.3 M) H_2_SO_4_ (96%, OM Group ultra-pure chemicals, Cleveland, OH, USA) at room temperature (20–25 °C) without agitation [10]. In order to investigate the impact of bath deposition temperature on the prepared thin films, the same EMD deposition process was performed in a bath heated to a temperature of 40 °C. All deposition experiments and electrochemical measurements were conducted using Autolab PGSTAT 30 (Metrohm, Utrecht, The Netherlands) or PGSTAT 100 potentiostats (Metrohm, Utrecht, The Netherlands). A three-electrode cell was established with Pt mesh (52 mesh woven from 0.1 mm (0.004 in) diameter wire, 99.9% (metals basis), 25 mm × 25 mm, supplied by Alfa Aesar Aesar, Karlsruhe, Germany) as a counter electrode (CE) and Ag/AgCl/3 M NaCl reference electrode (RE) (BASi analytical, 0.21 V vs. SHE). In what follows, all potentials are referred to the Ag/AgCl electrode unless otherwise noted. The working electrodes (WE) were C/TiN silicon pillar arrays. The area of the WE exposed to the electrolyte was 1 cm^2^. Before deposition, ohmic contact was made on the back side of the samples by Indium–Gallium (In–Ga).

### 2.4. Physical and Electrochemical Characterization of EMD Thin Films

After deposition, the water content in the EMD films was removed by thermal annealing at 350 °C in N_2_ atmosphere (200 mbar) for 3 h in a vacuum oven (+20 min. ramp-up). The electrochemical activity of EMD films was investigated by cyclic voltammetry (CV) at a scan rate of 10 mV/s between 2 and 4 V vs Li^+^/Li. CV measurements were carried out using a three-electrode Teflon cell in an argon-filled glove box (<1 ppm O_2_ and H_2_O levels). The substrates with EMD films were connected as the working electrode in the same way as for the deposition. Two ribbons of Li metal were used as reference and counter electrodes, respectively. The Li^+^ electrolyte solution was made up of 1 M LiClO_4_ (99.99%, Sigma-Aldrich, Overijse, Belgium) in Anhydrous Propylene Carbonate (99.7%, Sigma-Aldrich, Overijse, Belgium) solvent. 

The morphology of EMD films was checked by scanning electron microscopy (SEM) Philips-XL30 ESEM (FEI, Hillsboro, OR, USA).

## 3. Results and Discussion

### 3.1. Conformal Electrodeposition of EMD Films on C-Coated TiN/Si Pillars

**Figure 3:**
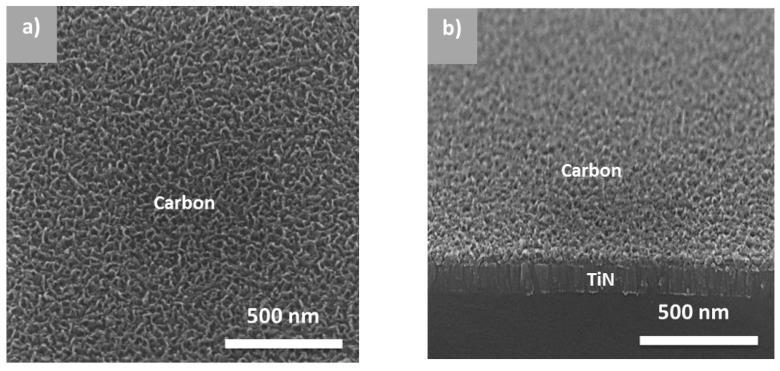
(**a**) Top and (**b**) cross-section SEM view of PECVD-carbon layer on 70 nm TiN/Si substrate.

Prior to EMD deposition, the benefit of thin carbon films as protective coatings for TiN diffusion barriers on silicon substrates was reported on planar substrates [52]. Carbon coatings with a nano-roughness of a few tens of nanometres were deposited by a PE-CVD process used for the growth of carbon nanosheets (CNS) [58]. In this case, the process was interrupted after the sheet nucleation step resulting in nano-rough carbon films of 30–50 nm thick as depicted in Figure 3a,b.

**Figure 4:**
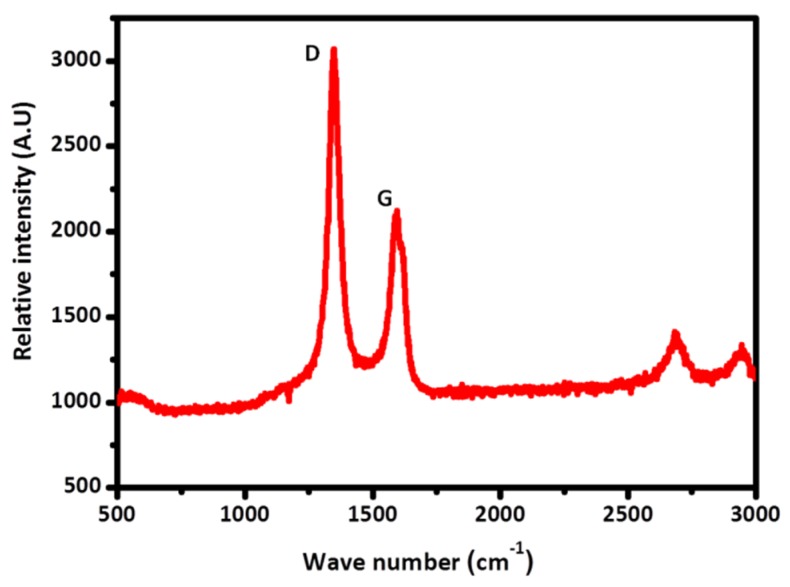
Raman spectrum for ~30–50 nm PECVD carbon grown on 70 nm TiN coated silicon substrate at 800 °C for 3 min.

Further analysis of the as-prepared PE-CVD carbon layer was performed by Raman spectroscopy. As illustrated in Figure 4, the Raman spectrum shows intense bands between 1360 and 1600 cm^−1^.

This carbon coating is a mixture of amorphous carbon (a-C) and graphitic carbon, as reported by Cott et al. [57]. The combination of an intense D-band (bandwidth ~45 cm^−1^) and a lower G-band (~59 cm^−1^) with a shoulder at 1620 cm^−1^ indicates the presence of defective graphitic (sp^2^ bonded) carbon. The broad component in the range 1300–1650 cm^−1^ can be assigned to a-C [59]. This composition of carbon offers a twofold benefit for EMD deposition. The a-C protects the TiN seed layer from oxidation as demonstrated in Ref. [52], while the graphitic portion improves the electrical conductivity of EMD [60].

For Li-ion battery applications, the crystalline carbon is not preferred, as the grain boundary has a low Li^+^ diffusion energy barrier and provides a fast Li^+^ diffusion [60]. Consequently, Li^+^ can be adsorbed at the grain boundaries [61,62]. Thus, the electrochemical performance of the electrode stack can be affected. This is why it is interesting to use such a coating with a combination of amorphous and graphitic carbon.

**Figure 5:**
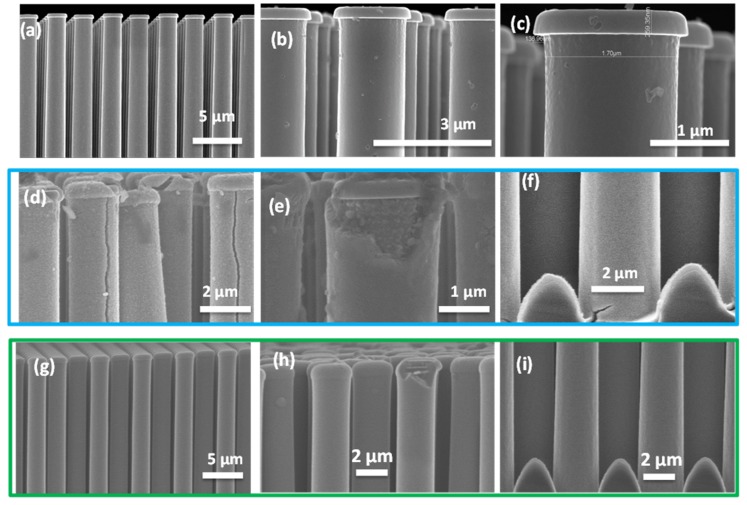
Scanning electron microscopy images showing the TiN silicon pillars (**a**–**c**) before carbon and EMD deposition. Cross-sectional SEM images for EMD anodically deposited on carbon-coated TiN/Si pillars under 4.5 mA/cm^2^ (effective current 0.2 mA/cm^2^) for 100 s at room temperature of 22 °C; (**d**–**f**) and at a bath temperature of 40 °C; (**g**–**i**). All EMD films were deposited from an aqueous bath of MnSO_4_ (0.3 M) in H_2_SO_4_ (0.3 M), without agitation.

After carbon and EMD deposition, the morphology of the coated high aspect ratio TiN/silicon pillars (Figure 5a–c) was investigated by SEM. Figure 5d–f show cross-sectional SEM images of EMD thin films anodically deposited on the carbon coated silicon pillars from an aqueous bath of MnSO_4_ (0.3 M) in H_2_SO_4_ (0.3 M) at room temperature (22 °C), without stirring, for 100 s.

The obtained SEM images illustrate conformal and homogenous EMD coatings on the 3D current collector structure. This morphology is due to the effectiveness of the carbon layer on TiN/Si pillars as well as self-limiting electrodeposition of MnO_2_ active material. A thick film of 230 nm covers the carbon-coated silicon from top to bottom as exhibited in Figure 5e,f. However, Figure 5d shows some cracks in EMD coating that can be explained by induced internal stresses in its structure. By increasing the bath deposition temperature to 40 °C, the morphology of EMD was improved as it can be seen for the SEM images in Figure 5g–i. The cracks previously seen disappeared and the conformal aspect of the coating is kept. However, the thickness of the deposited film is slightly increased to 240 nm compared to the one deposited at room temperature. In fact, the bath deposition temperature influences the morphology and structural properties of MnO_2_ deposited by electrolysis as reported by Ghaemi et al. [63].

Under the different deposition studied conditions, EMD film thickness was measured by SEM on top of the pillars (where the layer was broken off due to the cleaving of the sample), in the planar field area next to the pillar array and at the bottom of the pillars. No appreciable difference in EMD thickness was measured proving the excellent conformity of the films. 

**Figure 6:**
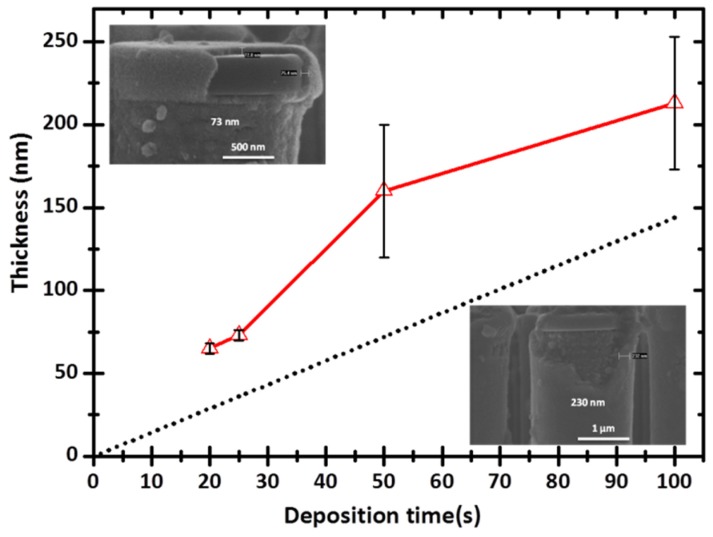
Theoretical thickness (**dotted line**) and experimental thickness (**full line**) vs. deposition time for EMD deposited at 4.5 mA/cm^2^ on carbon coated 3D pillar arrays with an area enhancement of 25×.

Figure 6 shows the variation of the average film thickness with deposition time together with the expected thickness in the case of dense MnO_2_ thin films based on Faraday’s law of electrolysis:

(1) $$Theoritical\;thickness=\frac{{\mathrm{MW}}_{{\mathrm{MnO}}_{2}}\times i_{d}\times t}{F\times n\times \rho_{{\mathrm{MnO}}_{2}}}$$

where MW_MnO_2__ = 86.93 g/mol is the molecular weight of MnO_2_, *i_d_* the applied current density in A/cm^2^, *t* is the deposition time in seconds (s), *F* = 96,485 C/mol is Faraday’s constant, *n* = 2 the number of electrons exchanged during the oxidation reaction and *ρ*_MnO_2__ = 5.03 g/cm^3^ is the density of MnO_2_. 

As depicted in Figure 6, the film thickness increases linearly with deposition time up to 1 min. It should be noted that the difference between theoretical thickness and actual measured EMD film thickness is of factor two as found for planar films with the expected porosity of ~50% [51,52]. This discrepancy in the film thickness is explained by the fact that EMD films are highly porous in nature. The porosity of MnO_2_ is important for charge transfer during deposition [64]. However, at high charge density, the effective current density drops, resulting in a subsequent drop in the degree of porosity.

The deviation from linearity for *t* > 50 s is likely due to a decrease in current efficiency when parasitic oxygen evolution reaction kicks in as reported in Ref. [51]. Moreover, high experimental thickness values could be attributed to the trapped water in EMD films structure during deposition as demonstrated by Johns et al. [65].

### 3.2. Electrochemical Lithiation/Delithiation Properties of EMD Deposited on C/TiN/Si Pillars

The electrochemical behavior of the annealed EMD films, at 350 °C in N_2_ atmosphere, was examined by cyclic voltammetry in 1 M LiClO_4_/PC battery electrolyte between 2 and 4 V vs. Li^+^/Li.

**Figure 7:**
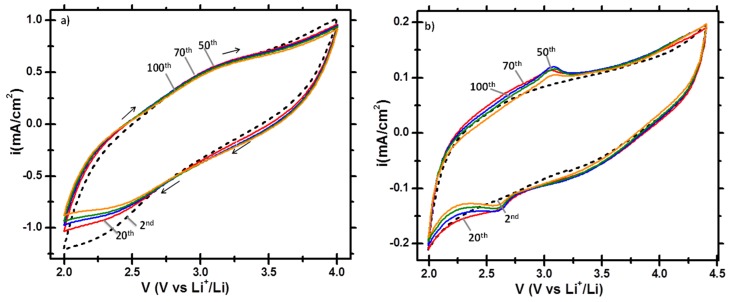
Cyclic voltammograms for Li-ion insertion (cathodic) and extraction (anodic) for (**a**) 230 nm EMD and (**b**) 70 nm EMD anodically deposited on C/TiN/Si pillars obtained in 1 M LiClO_4_/ propylene carbonate at a scan rate of 10 mVs^−1^. Cycles 2, 20, 50, 70 and 100 are shown for a total of 100 consecutive cycles. The EMD films were deposited at 4.5 mA/cm^−2^ and annealed at 350 °C in N_2_ atmosphere for three hours.

Figure 7a shows the recorded voltammograms of 230 nm-thick EMD film deposited on C/TiN/Si pillars at room temperature. The second cycle did not show any reversible current peaks for lithium ions insertion/extraction. However, a purely capacitive wave current response was observed. This poor electrochemical response is likely due to a decrease in the electrolyte access to the film, as well as an increase in the electronic resistance [66]. Upon cycling, the peak current slightly increases to reach its maximum by the 70th cycle and drops again as seen in the 100th cycle. This behavior highlights the impact of the overall resistance resulting in ohmic drop (IR) that affects the electrode performance. In this case, EMD coatings with such properties can be successfully used for electrochemical capacitors [67,68,69].

Figure 7b illustrates the voltammograms of 70 nm EMD deposited on C/TiN/Si pillars. The film was also not electrochemically activated at the second cycle. At a scan rate of 10 mV/s, Li^+^ does not have enough time to diffuse inside the film at the beginning of cycling. However, the magnitude of the peak current was relatively increased upon cycling. This could be attributed to improvement in Li^+^ intercalation kinetics of the EMD film. Small broad peaks appeared from the 10^th^ cycle at 2.6 and 3.05 V vs. Li^+^/Li, respectively. This electrochemical response of EMD thin-films is in agreement with previous findings [10,70,71]:

(2) $${\mathrm{MnO}}_{2}+x{\mathrm{Li}}^{+}+xe^{-}⇌{\mathrm{Li}}_{x}{\mathrm{MnO}}_{2}$$

The insertion of Li^+^ was accompanied with the reduction of Mn(IV) to Mn(III) in the EMD films. In the reverse scan, the Li^+^ are extracted upon the re-oxidation of Mn(III) to Mn(IV) [51]. The improved Li^+^ diffusion, in 70 nm EMD film, can be explained by several factors. For instance, the decreased thickness implies a decrease in the overall resistance. This consequently compensates for the IR drop and leads to increasing the film porosity. However, the electrochemical activity faded after the 50th cycle for the 230 nm EMD film. Side reactions at the electrode/liquid electrolyte interface as well as Li^+^ diffusion kinetics limitations might suppress the activity of EMD thin film [72,73]. Furthermore, the composition of the film could be influenced by the deposition conditions of electrodeposition as time and charge density. Consequently, the percentage of each species within the heat-treated EMD has an effect on its electrochemical performance. Dose et al. reported that the relationship between Mn(IV) and Mn(III) is inversely proportional, as low Mn(IV) entails high Mn(III), resulting in a decrease in the performance of the electrode [74,75]:

(3) $${\mathrm{Mn}}^{IV}O_{2}+xe^{-}+x{\mathrm{Li}}^{+}⇋{\mathrm{Li}}_{x}{\mathrm{Mn}}_{x}^{III}{\mathrm{Mn}}_{1-x}^{IV}O_{2}$$

**Figure 8:**
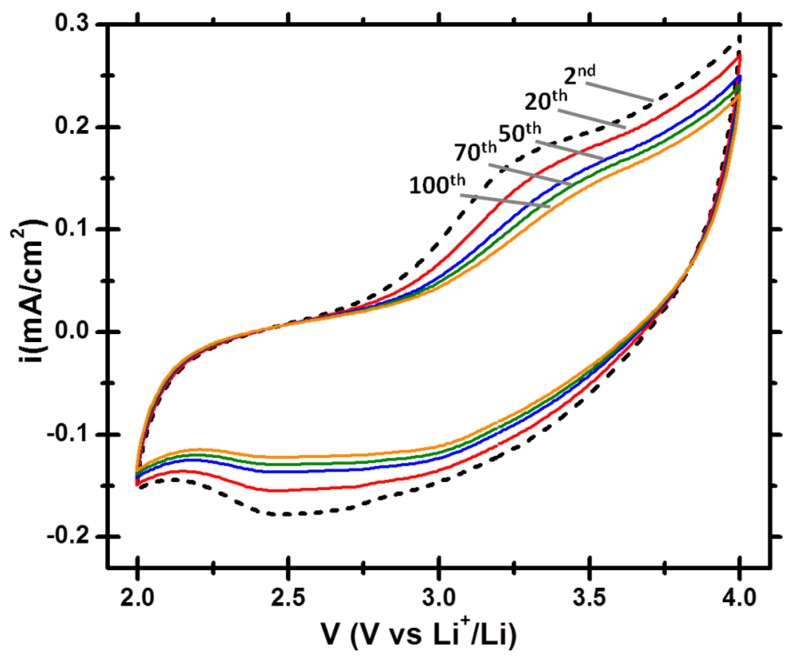
Cyclic voltammograms for Li-ion insertion and extraction for 240 nm EMD anodically deposited on C/TiN/Si pillars obtained in 1 M LiClO_4_ in propylene carbonate at a scan rate of 10 mVs^−1^—Cycles 2, 20, 50,70 and 100 are shown for a total of 100 consecutive cycles. The EMD films were deposited at 4.5 mA/cm^2^ s at 40 °C. After the deposition, the annealing step is made at 350 °C for three hours in N_2_ atmosphere to enable the lithiation.

Figure 8 presents the recorded voltammograms of 240 nm EMD deposited on C/TiN/Si pillars at 40 °C. These measurements had a goal of making use of the conformal thick film deposited over carbon coated pillar arrays while evaluating the influence of bath deposition temperature on its electrochemical performance.

Perversely to the EMD film prepared at room temperature, the deposited film at 40 °C exhibited a capacitive current response from the second cycle with small broad peaks of electrochemical activity at 2.5 and 3.25 V vs. Li^+^/Li, respectively. However, the electrochemical performance faded upon cycling. This is likely due to Li^+^ diffusion limitations and/or to the remaining water content in EMD [65], resulting in the formation of secondary products like MnOOH [38]. Such a product could be dissolved, releasing Mn(III) ions to the electrolyte and reduced to Mn(II) species later on [76]. This partial dissolution of EMD films into the electrolyte during cycling can explain the poor electrochemical behavior noted in Figure 8. The use of additives, such as TiB_2_, to the deposited film and the optimization of the heat-treatment before lithium intercalation are recommended [76,77].

## 4. Conclusions

In summary, we have achieved conformal EMD films on high aspect ratio carbon coated TiN/Si pillars by means of electrochemical deposition (ECD). The use of a thin carbon buffer layer on 3D TiN/Si pillar arrays has been found to be advantageous due to its composition consisting of graphitic and amorphous carbon enabling the deposition of γ-MnO_2_ on the 3D current collector stack with good adhesion and without the oxidation of the underlying ALD TiN layer. The electrochemical performance of the deposited films, in terms of Li^+^ insertion/extraction, depends on γ-MnO_2_ characteristics such as thickness and porosity. Even though no apparent morphological differences were found between EMD films prepared at different bath temperatures, their electrochemical activity implies that the composition might not be the same. Therefore, further optimization for the post-deposition thermal treatment should be addressed to get more insights on the relationship between the compositional changes of EMD films and their electrochemical behavior on the 3D C/TiN/Si pillars.

## Supplementary Materials

Supplementary File 1
