# Supplementary material for: Electrolytic Manganese Dioxide Coatings on High Aspect Ratio Micro-Pillar Arrays for 3D Thin Film Lithium Ion Batteries
Source: Nanomaterials (Basel). 2017 May 27;7(6):126. doi: 10.3390/nano7060126 (PMC5485773; doi:10.3390/nano7060126)
Supplement: Supplementary File 1 [file nanomaterials-07-00126-s001.pdf]

Supplementary Materials: Electrolytic Manganese Dioxide Coatings on High Aspect Ratio Micro-Pillar Arrays for 3D Thin Film Lithium Ion Batteries

Yafa Zargouni [1,2,3,4,*], Stella Deheryan [2,4], Alex Radisic [2], Khaled Alouani [3] and Philippe M. Vereecken [2,4]

EDX analysis using NOVA 200 Microscope.

Figure S1a and b show the EDX spectra for the as-deposited γ-MnO_2 film carried out on different points of the sample. The presence of manganese and oxygen peaks confirm that the deposited material on carbon coated TiN/Si pillars is a manganese oxide. The morphology of the tested sample is also illustrated in SEM images on the left of EDX spectra in figure 1.

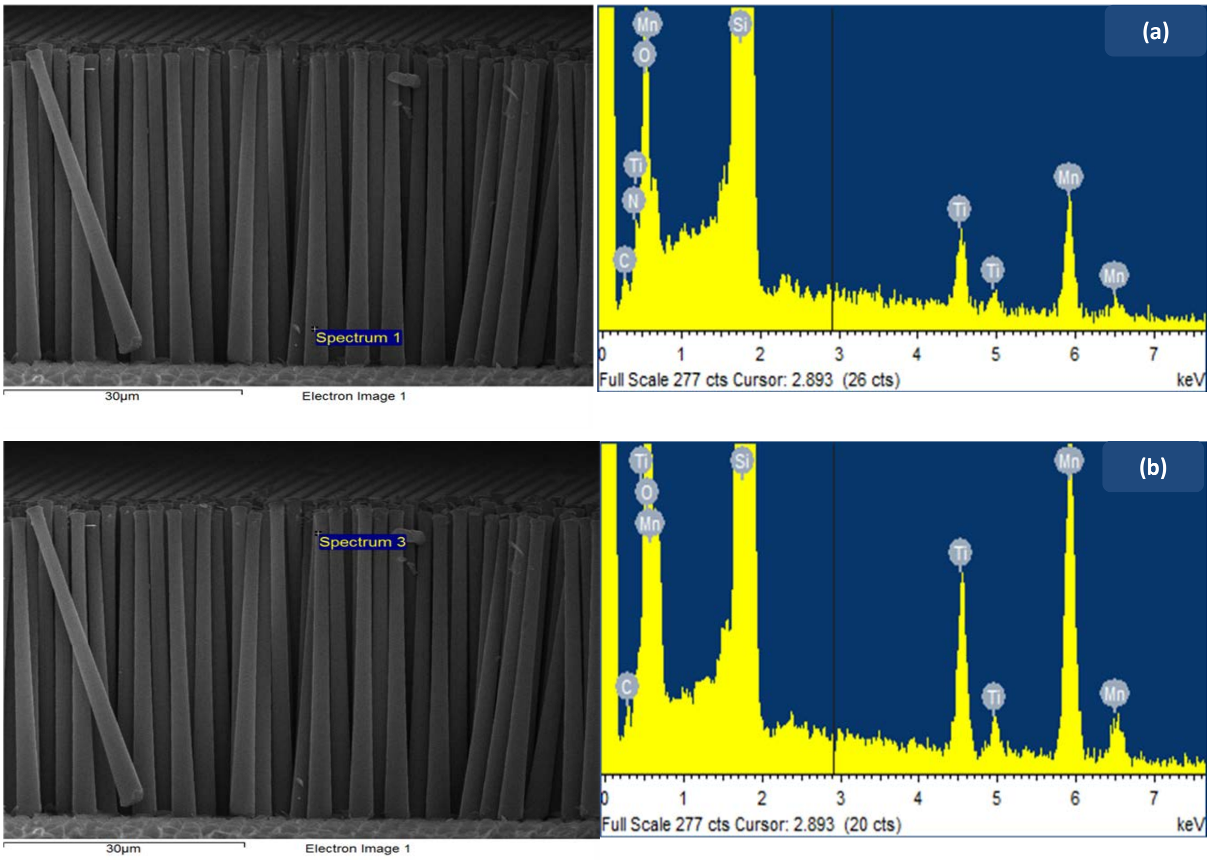

Figure S1. EDX spectra of as-deposited γ-MnO_2 on carbon coated TiN/Si pillars.
